# Fludarabine and rituximab with escalating doses of lenalidomide followed by lenalidomide/rituximab maintenance in previously untreated chronic lymphocytic leukaemia (CLL): the REVLIRIT CLL-5 AGMT phase I/II study

**DOI:** 10.1007/s00277-018-3380-z

**Published:** 2018-06-04

**Authors:** Alexander Egle, Michael Steurer, Thomas Melchardt, Lukas Weiss, Franz Josef Gassner, Nadja Zaborsky, Roland Geisberger, Kemal Catakovic, Tanja Nicole Hartmann, Lisa Pleyer, Daniela Voskova, Josef Thaler, Alois Lang, Michael Girschikofsky, Andreas Petzer, Richard Greil

**Affiliations:** 1 Department of Internal Medicine III with Hematology and Medical Oncology, Oncologic Center, Paracelsus Medical University Salzburg, Müllner-Hauptstr. 48, 5020 Salzburg, Austria; 2 Salzburg Cancer Research Institute - Laboratory of Immunological and Molecular Cancer Research (SCRI-LIMCR) and Cancer Cluster Salzburg (CCS), Salzburg, Austria; 3 Division of Hematology and Oncology, Laboratory for Molecular Genetics and Diagnostics, Innsbruck Medical University, Innsbruck, Austria; 4 Centre for Hematology and Medical Oncology, Kepler University Hospital, Linz, Austria; 5 Department for Internal Medicine IV, Hospital Wels-Grieskirchen, Wels, Austria; 6 Internal Medicine, Hospital, Feldkirch, Austria; 7 Internal Medicine I (Hemostasis, Hematology and Stem Cell Transplantation and Medical Oncology), Ordensklinikum Linz Elisabethinen, Linz, Austria; 8 Department for Internal Medicine IV, Hospital Barmherzige Schwestern Linz, Linz, Austria

**Keywords:** CLL, Lenalidomide, Rituximab, Fludarabine, Combination

## Abstract

Despite recent advances, chemoimmunotherapy remains a standard for fit previously untreated chronic lymphocytic leukaemia patients. Lenalidomide had activity in early monotherapy trials, but tumour lysis and flare proved major obstacles in its development. We combined lenalidomide in increasing doses with six cycles of fludarabine and rituximab (FR), followed by lenalidomide/rituximab maintenance. In 45 chemo-naive patients, included in this trial, individual tolerability of the combination was highly divergent and no systematic toxicity determining a maximum tolerated dose was found. Grade 3/4 neutropenia (71%) was high, but only 7% experienced grade 3 infections. No tumour lysis or flare > grade 2 was observed, but skin toxicity proved dose-limiting in nine patients (20%). Overall and complete response rates after induction were 89 and 44% by intention-to-treat, respectively. At a median follow-up of 78.7 months, median progression-free survival (PFS) was 60.3 months. Minimal residual disease and immunoglobulin variable region heavy chain mutation state predicted PFS and TP53 mutation most strongly predicted OS. Baseline clinical factors did not predict tolerance to the immunomodulatory drug lenalidomide, but pretreatment immunophenotypes of T cells showed exhausted memory CD4 cells to predict early dose-limiting non-haematologic events. Overall, combining lenalidomide with FR was feasible and effective, but individual changes in the immune system seemed associated with limiting side effects. clinicaltrials.gov (NCT00738829) and EU Clinical Trials Register (www.clinicaltrialsregister.eu, 2008-001430-27)

**Electronic supplementary material:**

The online version of this article (10.1007/s00277-018-3380-z) contains supplementary material, which is available to authorized users.

## Introduction

Treatment of chronic lymphocytic leukaemia (CLL) in previously untreated patients has improved remarkably over the past 10 to 15 years [1]. Specifically, in fit patients, chemoimmunotherapy combining fludarabine, cyclophosphamide and rituximab remarkably increased overall survival [2] and became a standard of care for induction. However, toxicities may limit this approach to younger and fitter patients. In addition, recently gained insights into the pathobiology of CLL suggest that targeted treatment of CLL may be feasible by either attacking specific intracellular pathways or using substances that modify the microenvironment more globally [3]. Examples of the former (such as ibrutinib, idelalisib, or venetoclax) have now entered the clinical field in relapsed/refractory patients [4, 5] and have also entered initial therapy. While ibrutinib is licenced for the treatment of previously untreated patients regardless of the physical fitness, the use of idelalisib/rituximab or venetoclax in front-line treatment is restricted to CLL patients with del17p that are not suitable for other therapies. Among the options for microenvironment modification, lenalidomide has attracted interest for a number of years (reviewed in Kater et al.) [6].

Initial findings of efficacy of lenalidomide [7, 8] were accompanied by reports of so-called tumour flare reactions and a number of observed and sometimes life-threatening tumour lysis reactions [7, 9]. Since these observations were made at the 25-mg dose, previously established for multiple myeloma [10, 11], this led to a reassessment of lenalidomide dosing in CLL and the development program for the substance mandated a dosing schedule following slowly increasing doses in trials started from 2007 on. In monotherapy, this schedule proved tolerable [12], but showed much less efficacy than expected from trials presented with higher doses [7, 8].

A combination of a chemoimmunotherapy backbone (fludarabine and rituximab) with lenalidomide may offer a treatment option that allows the use of three effective principles with different mechanisms of action. In addition, we hypothesised that an initial phase of chemoimmunotherapy for the debulkment of CLL might reduce or even eliminate lenalidomide-induced tumour lysis by reducing the tumour burden and possibly decrease the probability of tumour flares. At the same time, this triple combination may allow a slow increase in lenalidomide doses to a maximum tolerable dose without running the risk of missing lenalidomide efficacy due to slow response and early progress. A further question was the feasibility of a prolonged treatment period with lenalidomide and rituximab after remission induction with chemoimmunotherapy plus lenalidomide.

We designed the RevliRit trial to (a) determine individual maximum tolerated doses for lenalidomide in combination with fludarabine and rituximab; (b) explore toxicity and efficacy of the regimen, also using a panel of parallel laboratory investigation for exploratory analyses and (c) to determine the feasibility of a lenalidomide/rituximab consolidation/maintenance treatment.

## Patients and methods

This study protocol was approved by the ethics committee of the state of Salzburg on July 30, 2008 (protocol number: 415-E/966) and by the ethics committee of each individual participating centre.

All patients had given their written informed consent. Patients with previously untreated CLL were enrolled onto this phase I/II trial. Patients had a diagnosis of CLL and active disease as defined by the 1996 NCI WG criteria [13]. Further inclusion criteria consisted of age older than 18 years, ECOG performance status 0–2 and understanding of the need of effective contraception. Exclusion criteria were positivity for HIV or hepatitis B or C, other active infections, a creatinine clearance of below 30 ml/min and a history of other malignancy within the previous 2 years, with exception of localised skin, cervix, prostate and breast cancer, likely to be cured by surgery. Also excluded were patients with severe heart disease or other conditions severely limiting life expectancy.

### Study design and treatment

This was a non-randomised, multicentre, open-label, single-arm phase I/II trial of the Arbeitsgemeinschaft Medikamentöse Tumortherapie (AGMT) trial consortium. All patients were included in this trial at seven cancer centres of the AGMT between September 2008 and November 2010. The phase I part evaluated the maximum tolerated lenalidomide dose level in combination with fludarabine and rituximab (FR) chemoimmunotherapy. Phase II of the study was planned to determine efficacy using the combination with the previously defined maximum tolerated dose (MTD) as plateau dose for dose escalation. An induction phase was followed by a maintenance phase evaluating the tolerability and possibility to further improve response quality (Supplementary Fig. S1).

In the induction phase, an MTD of lenalidomide in combination with FR was to be determined during six cycles of fludarabine (40 mg/m^2^ per os (po) days 1–3, repeated every 4 weeks) and rituximab (375 mg/m^2^ intravenously (iv) day 4 at cycle 1; 500 mg/m^2^ iv day 1 at cycles 2–6, repeated every 4 weeks). In cycle 1, lenalidomide was added from days 8 to 21 at 2.5 mg. Toxicity permitting, lenalidomide dose was escalated to 5, 10, 15, 20 and 25 mg on days 1–21 over cycles 2–6. Dose-limiting toxicities were defined as serious infections or limiting grade 3/4 toxicities, other than neutropenia. The latter was excluded from the definition of dose-limiting toxicities (DLT) because the FR backbone was expected to lead to a significant rate of grades 3–4 neutropenia by itself [14]. However, prolonged neutropenia (neutropenia not resolving to grade ≤ 2 within 4 weeks) was defined as DLT. Patients were allowed to continue on the last tolerated dose or enter a stepwise de-escalation should a new DLT appear at a previously tolerated dose level. Because of a notion of an individual component in lenalidomide tolerability in reported trials [7, 8, 12], an individual per patient dose escalation was favoured over a 3 + 3 design for dose escalation. Initially, the stepwise lenalidomide escalation was planned for the first 10 patients in the trial, using 24-h reporting of all grade 3/4 adverse events (AEs) as a safety measure for these patients to define a MTD for the remainder of the trial. Due to the findings in the first 10 patients (see “Results” section), an amendment specified that all patients could be treated with the escalation schedule to their individual MTD.

In the maintenance phase, lenalidomide was planned to continue for six cycles continuously at the individually determined MTD, changing the schedule to a continuous 28-day cycle. De-escalation to a 21-day cycle and a stepwise dose de-escalation of lenalidomide were allowed for toxicity. Rituximab (375 mg/m^2^ iv) was administered after maintenance cycles 2, 4 and 6. For patients going off lenalidomide for any reason, completion of chemoimmunotherapy and rituximab maintenance was allowed for intention to treat (ITT).

Antimicrobial prophylaxis and growth factor support were not mandatory and were allowed per institutional practice. Antithrombotic prophylaxis with aspirin at a dose between 70 and 100 mg per day was mandated during lenalidomide treatment as an amendment after 10 patients.

### Study endpoints and assessment

The primary endpoint was to determine the MTD per patient of lenalidomide in combination with FR. Secondary endpoints were efficacy and safety, risk factor dependency and minimal residual disease (MRD) analyses as well as T cell subset analyses.

Clinical and laboratory assessment was performed before beginning of every cycle during induction and maintenance phase. Computed tomography scans to assess nodal disease and MRD analyses from peripheral blood were performed after three and six cycles of induction treatment and after three cycles and completion of maintenance. MRD analyses were performed in a central laboratory (LIMCR at the IIIrd Medical Department in Salzburg) using a predefined 4-colour panel (according to Rawstron 2001) [15] and using a cutoff of 10^−3^ to define MRD negativity. Response was assessed using the 1996 National Cancer Institute guidelines including computed tomography (CT) scan results and bone marrow cytology and histology [13]. Molecular risk assessment was performed in the central laboratory (LIMCR) as previously described [16]. All patients that had received at least one dose of the study medication have been followed for adverse events (AEs) for at least 28 days after discontinuing study treatment or completion of study treatment. AEs have been assessed according to Common Terminology Criteria for Adverse Events v3.0 (CTCAE) by the investigator.

### T cell analyses

T cell analyses were performed in purified peripheral blood mononuclear cells (PBMCs). A primary stain with unlabelled murine anti-human antibodies directed against PD-1 (clone EH12.2H7) or isotype control followed with secondary phycoerythrin (PE)-labelled goat-anti-mouse immunoglobulin antibody (Dako, Glostrup, Denmark) was followed by primary labelled surface stains as follows: CD4 (Pacific Blue); CD5 (allophycocyanin (APC)-AlexaFluor 700); CD25 (fluorescein isothiocyanate, FITC); CD45RA (energy-coupled dye, ECD or FITC); CD62L (PE or phycoerythrin-cyanin 5, PC5); CD127 (PE) (all from Beckman Coulter, CA, USA); CD8 (Pacific Orange) (Invitrogen, MD, USA); CD183 (APC) and CD194 (phycoerythrin-cyanin 7, PC7) (all from BD Biosciences, CA, USA). Analyses were done on a Gallios flow cytometer and T cell subsets were defined as follows: naïve (CD62L+CD45RA+), central memory (CM, CD62L−CD45RA+), effector memory (EM, CD62L−CD45RA−), TEMRA (CD62L+CD45RA−), TH1 (CD183+CD194−), TH2 (CD183−CD194+) and Treg (CD25+CD127−). Statistical analyses were performed using unpaired *t* tests for all subgroup results. A simple Bonferroni correction was applied to adjust for multiple testing, leading to a necessary *p* value of < 0.0025 for the definition of significance.

### Gene mutation analyses

#### Patients and samples

CLL cells were isolated from PBMCs using a B-CLL Cell Isolation Kit (Miltenyi Biotec). Cell purity was > 90% in all samples. DNA was purified using a DNeasy blood and tissue kit (QIAGEN). Libraries were enriched using Agilent SureSelectXT Human All Exon V5 Kit +UTRs and sequenced on a HiSeq 2000 using 100-bp paired end reads by GATC Biotech AG, Konstanz, Germany, with a mean coverage depth of ×60.

#### Bioinformatic analysis

Mapping to human reference genome (hg19) (bowtie2 (v2.1.0)) and quality adjustments were performed using PicardTools (v2.2.2, http://broadinstitute.github.io/picard/) and Genome Analysis Tool Kit (GATKv3.5) [17]. After mpileup generation by samtools (v1.3.1) [18], VarScan2 (v2.3.7) [19] and fpfilter.pl script were used for somatic variant calling and filtering of high confidence calls. From annotated variants (ANNOVAR (version 2015 Dec14) [20]), only non-synonymous exonic or splice-site variants as well as NOTCH1 3′ UTR variants were considered and “non-deleterious” (SIFT, PolyPhen, MutationTaster) or “non-pathogenic” (ClinVar, COSMIC) and established single-nucleotide polymorphisms were filtered out. All remaining mutations were manually checked for artefacts using the Integrative Genomics Viewer (IGV) [21, 22].

Mutations were evaluated for a set of genes that has been chosen, because there were published datasets suggesting prognostic or predictive impact of these mutated genes. The genes were *ATM* [23], *BIRC3* [23], *NFKBIE* [24], *NOTCH1* [25], *SF3B1* [26], *TP53* [23, 26], ZFN292 [23] and *RPS15* [26]. No mutations were found in the latter.

### Statistical analyses

Statistical analyses were performed using IBM® SPSS® statistics software, version 24. Survival was estimated using Kaplan-Meier curve analysis, with statistical comparison using the log-rank statistic. For progression-free survival (PFS), analysis events were progression or death to any reason. Study dropouts were censored at the time of dropout. A two-tailed significance level of 0.05 was considered statistically significant. Cox regression was used for univariate analyses, where appropriate. Median follow-up was calculated by the reverse OS method using Kaplan-Meier curve analysis.

## Results

### Patient characteristics and patient disposition

The median age of the 45 patients included was 66 years (range 43–79) and 58% were male. Fifty percent of the patients presented with Rai stage III/IV and 7 and 22% harboured del17p or del11q, respectively. Further, a majority of 86% showed mutations in any one of eight genes previously reported to be associated with clinical high-risk behaviour [23–26]. Regarding comorbidities, the median creatinine clearance was 72 ml/min. We have recorded a total of 183 comorbidities recorded in 41 of our patients and 80 of these comorbidities had active medication at the start of the trial. Overall, this suggests a population that was less fit than one generally included in an fludarabine, cyclophosphamide and rituximab (FCR) trial. Patient characteristics are outlined in Table 1.

**Table 1:** Patient characteristics

Baseline characteristics	
Median age, years (range, IQR)	66 (43–79, 61–70)
Leucocytes, ×10^9^ cells/L (range, IQR)	94.6 (11–374, 49.3–149)
Creatinine clearance, ml/min (median, range, IQR)	72.5 (32.5–141.7, 61.5–91.6)
Risk factors	
Rai stage III/IV	22/44 (50%)
B symptoms	17/45 (38%)
FISH cytogenetic (highest risk)	
Del(13q)	19/45 (42%)
Trisomy 12	7/45 (16%)
Del(11q)	10/45 (22%)
Del(17p)	3/45 (7%)
No cytogenetic abnormalities	6/45 (13%)
Gene mutations	
*ATM*	20/42 (48%)
*BIRC3*	2/42 (5%)
*NFKBIE*	6/42 (14%)
*NOTCH1*	13/42 (31%)
*SF3B1*	4/42 (9%)
*TP53*	3/42 (7%)
*ZNF292*	7/42 (16%)
Unmutated IgVH	21/38 (55%)
β2 microglobulin ≥ 3.9 mg/L	28/44 (64%)

The initial phase of the trial was designed to find an MTD of lenalidomide in the first 10 patients. This dose was then to be used as the plateau dose for dose escalation for the remainder of the trial. These first patients were recruited in two centres only and intense monitoring of AEs was performed. However, individual tolerability was highly variable. While patient number 1 could proceed to the maximal dose of 25 mg lenalidomide/day during induction treatment without experiencing limiting toxicities, patient number 2 stopped at 5 mg due to a rash and stopped lenalidomide after the rash returned at 2.5 mg. Altogether, in the first 10 patients, we observed an almost binary tolerability of the drug in combination, with 4 patients escalating to 25 mg without problems, while 5 patients had tolerated a maximum of 5 mg or less. Due to this apparent individual component of tolerability, the protocol was amended to allow individual dose finding in all patients. Notably, no life-threatening DLTs had been observed.

**Fig. 1:**
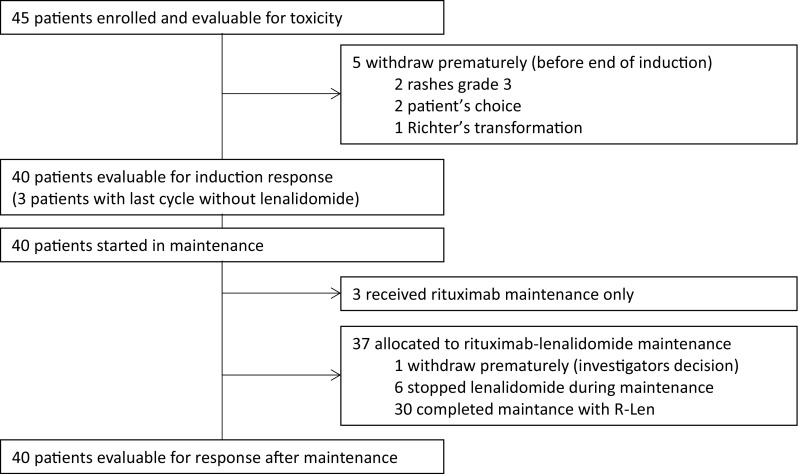
CONSORT diagram of patient disposition throughout the trial. Patient disposition is shown and further explained in the text

The overall disposition of patients was as follows (Fig. 1): Toxicity assessments of all 45 patients enrolled were evaluable. Five patients (11%) withdrew from the study during the induction period: two due to skin rashes grade 3, two due to patient’s wish and one patient due to a Richter’s transformation that seemed to have already been present at study inclusion in hindsight. Consequently, 40 patients were available for assessment of induction response, with 3 of these receiving the last induction cycle without lenalidomide and therefore entering maintenance receiving only rituximab. Of the 37 patients entering combination-maintenance, 30 finished with the combination and all 40 patients entering maintenance were evaluable for response after maintenance.

### Overall results of the dose escalation study

**Fig. 2:**
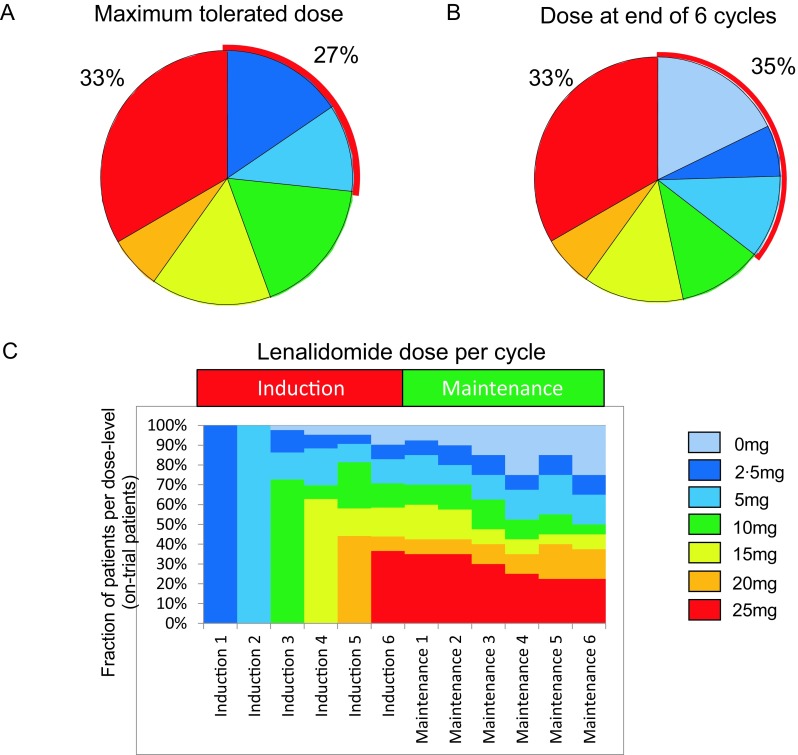
Achievable lenalidomide doses in combination treatment throughout the trial. **a** Maximum tolerated doses in induction combination with fludarabine and rituximab established by individual per patient dose finding. **b** Dose administered with the last of six cycles of FR (includes dose reductions after two stable cycles at the established MTD). **c** Fraction of on-trial patients per individual dose level per cycle of treatment

Regarding the primary endpoint, no systematic toxicity determining an MTD of lenalidomide was found in induction treatment in combination with FR, as was already suspected from the results in the 10 run-in patients. When the individual MTD was determined as tolerated dose for at least two consecutive cycles, the individual MTD was 2·5, 5, 10, 15, 20 or 25 mg in 16, 11, 18, 15, 7 and 33% of the patients, respectively (Fig. 2a). Still, some further dose modifications were performed per protocol in individual patients after two stable cycles, mostly due to prolonged cytopenias. Thus, by the end of six cycles, 18% of the ITT population was without lenalidomide (including the 5 patients that went off study). By contrast, 33% of the patients finished cycle 6 with 25 mg lenalidomide (Fig. 2b), suggesting a greater than 1 log difference in tolerated dose between individual patients.

In the maintenance phase, the dose established as the individual MTD in chemoimmunotherapy combination should have been carried on in a continuous dosing schedule. However, dose reductions were necessary in 46% of patients (Fig. 2c), mainly due to prolonged neutropenia (see below), but 47% of patients received 10 mg or greater for 6 months of maintenance treatment. Interestingly, 9/13 patients receiving 25 mg as initial maintenance dose were able to receive the treatment uninterrupted for 6 months, suggesting that a biologically distinct group may tolerate very high doses for a prolonged period.

### Toxicity of induction

Toxicity assessment was evaluable in all 45 patients. Maximal haematological and non-haematological side effects per patient in all body systems are summarised in Table 2. Considering the fact that all patients were treated until a DLT appeared or until reaching 25 mg lenalidomide without DLT, it is not surprising that we observed a high rate of grade 3/4 toxicities. The most common side effect in the induction was grade 3/4 neutropenia observed in 71% of the patients, which is within the range reported for FR therapy in first-line patients [14]. However, grade 3 infections were only observed in 7% of the patients during the induction phase and this compares relatively well with results from patients treated with FCR in first line, with a reported rate of 19% grade 3/4 infections from the CLL8 trial [2]. As per institutional decision, 23/45 patients had received prophylaxis for pneumocystis jirovecii pneumonia (PJP) and 16 patients received antiviral prophylaxis. In addition, G-CSF support was used in 17 patients. Somewhat unexpectedly, the most common non-haematological side effect during induction treatment were skin toxicities (mostly rashes) occurring in 80% (36/45) of the patients when all grades were counted and in 6.7% (3/45) when grade 3 was reported. These rashes started early during treatment and even at low doses of lenalidomide. Clinical symptoms were mostly mild and topical treatment was sufficient in the majority of patients but rashes led to dose limitations in 6 patients (13%) and were therefore a major obstacle to dose escalation. No case of tumour lysis syndrome (TLS) was reported, but three cases showed asymptomatic grade 4 hyperuricemia during induction phase. Two cases of venous thrombosis were reported after the initial 10 patients, but no further event was observed after thromboembolic prophylaxis was amended. The overall rate of thrombosis was thus 4.4% and below previously reported rates [7, 27]. At higher doses of lenalidomide, prolonged cytopenias became a DLT with a total of 16 patients (35%) being dose-limited for haematological toxicity (mostly above doses of 15 mg lenalidomide) (Supplementary Fig. S2). Finally, 5 patients stopped dose escalation due to their own wish (mostly citing not further classified, non-specific general symptoms), two of which stopped further trial participation altogether.

**Table 2:** Haematological and non-haematological side effects*

Body system	Induction (*N* = 45)			Maintenance (*N* = 40)		
	G1/2, %	G3, %	G4, %	G1/2, %	G3, %	G4, %
Cardiac general	8.8	2.2	0	2.5	0	0
Constitutional symptoms	71.1	0	0	57.5	0	0
Dermatology/skin	71.1	6.7	0	42.5	2.5	0
Gastrointestinal	62.3	2.2	0	45	0	0
Hepatobiliary/pancreas	11.1	4.4	0	2.5	2.5	0
Infection	55.6	6.7	0	42.5	5	0
Lymphatics	20	0	0	5	0	0
Neurology	22.2	0	0	17.5	0	0
Pain	53.4	2.2	0	30	0	0
Pulmonary/upper respiratory (excl. infections)	8.8	0	0	2.5	0	0
Renal/genitourinary	35.5	0	6.7**	20	0	0
Other	73.3	2.2***	0	50	2.5	0
Blood/bone marrow (excerpt)						
Neutropenia	26.6	35.6	35.6	17.5	45	27.5
Thrombocytopenia	80	4.4	4.4	77.5	0	2.5
Anaemia	84.4	4.4	2.2	55	0	2.5

*Percentages refer to number of patients with their maximal CTCAE grading per body system

**Three patients had G4 hyperuricemia without >G2 creatinine increase (no TLS)

***One patient was documented with hypertension/worsening of hearing

In the maintenance phase, the major toxicity was neutropenia with 45 and 28% reaching grades 3 and 4, respectively. Surprisingly, this did not translate into a highly relevant signal for infections. Grade 3, but no grade 4, infections were observed in 5% of patients and all other grade 3/4 toxicities remained below 5%. Compared to the reported incidence of skin reactions in induction, we did not observe a significant signal in the maintenance phase (Table 2).

### Efficacy of induction

Efficacy analyses were performed as exploratory analyses in this trial. A total of 40 patients (89%) were eligible for response assessment after induction and after maintenance. The ORR after the end of induction therapy was 89% in an ITT analysis including 44% complete responses (CR). Table 3 shows clinical and MRD response data for evaluable patients by subgroup.

**Table 3:** Responses (clinical and MRD) by subgroups in evaluable patients

Group	Response after induction		MRD response after induction	
	CR	PR	MRD negative	MRD positive
All*	20/40 (50%)	20/40 (50%)	22/39 (56%)	17/39 (44%)
Age > 65	15/22 (68%)	7/22 (32%)	17/22 (77%)	5/22 (23%)
FISH cytogenetics (hierarchical risk)				
Del(13q)	11/18 (61%)	7/18 (39%)	8/17 (47%)	9/17 (53%)
Trisomy 12	3/7 (43%)	4/7 (57%)	5/7 (71%)	2/7 (29%)
Del(11q)	3/9 (33%)	6/9 (67%)	5/9 (56%)	4/9 (44%)
Del(17p)	1/2 (50%)	1/2 (50%)	1/2 (50%)	1/2 (50%)
No cytogenetic abnormalities	2/4 (50%)	2/4 (50%)	3/4 (75%)	1/4 (25%)
High-risk gene mutations				
*ATM*	11/19 (58%)	8/19 (42%)	10/19 (53%)	9/19 (47%)
*BIRC3*	1/2 (50%)	1/2 (50%)	2/2 (100%)	0/2 (0%)
*NFKBIE*	2/4 (50%)	2/4 (50%)	1/4 (25%)	3/4 (75%)
*NOTCH1*	6/11 (55%)	5/11 (45%)	8/11 (73%)	3/11 (27%)
*SF3B1*	3/3 (100%)	0/3 (0%)	3/3 (100%)	0/3 (0%)
*TP53*	1/2 (50%)	1/2 (50%)	1/2 (50%)	1/2 (50%)
*ZNF292*	4/6 (67%)	2/6 (33%)	1/6 (17%)	5/6 (83%)
Unmutated IgVH	11/17 (65%)	6/17 (35%)	10/17 (59%)	7/17 (41%)

*Forty patients evaluable for response; 39 patients with central MRD assessment; 75% of CRs were MRD negative

Response assessment after maintenance showed improvement of response in five patients (improvement from partial response (PR) after induction to CR after maintenance in 12%), but the additional effect of maintenance seems to have been offset by toxicities and study dropouts when ITT analysis was performed.

**Fig. 3:**
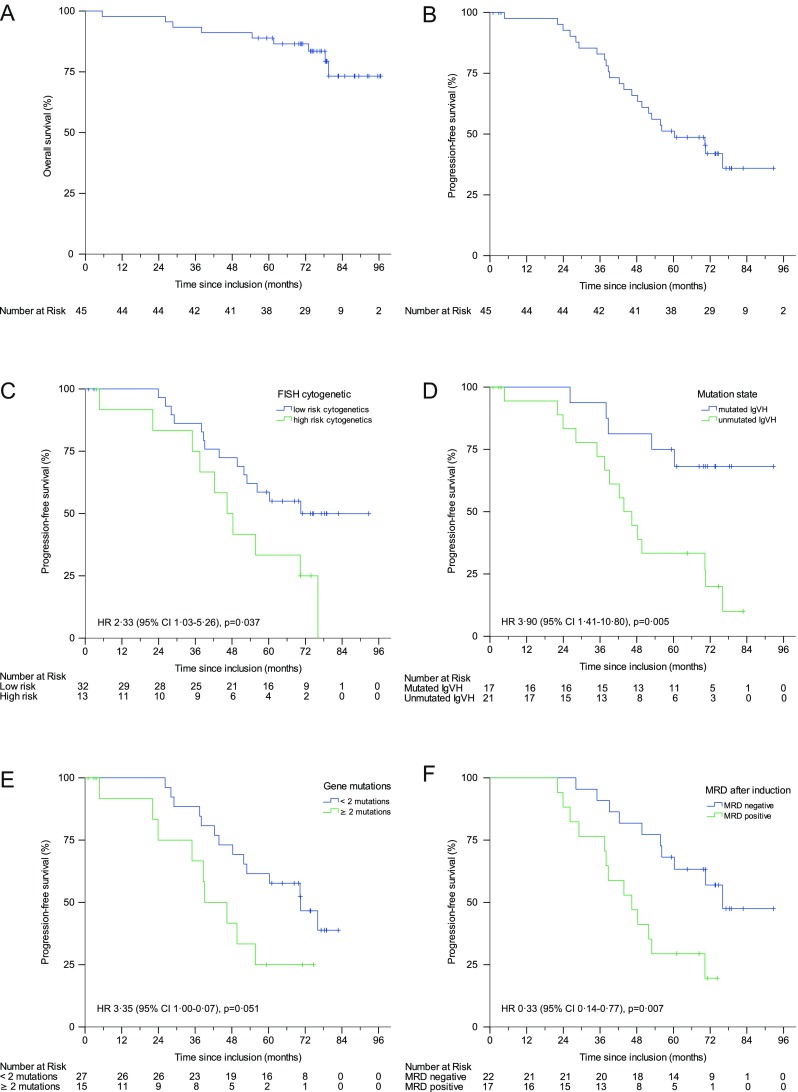
Kaplan-Meier estimates of **a** overall survival, **b** progression-free survival (PFS), **c** PFS by cytogenetic subgroup, **d** PFS by mutations state, **e** PFS by number of gene mutations and **f** PFS by MRD state after induction

The median follow-up in this analysis was 78.7 months (95% CI 76.7–80.7). Median PFS was 60.3 months (95% CI 41.3–79.3) and median OS was not reached. Five-year OS was 89% (Fig. 3a, b). Further exploratory analyses were performed to better understand response (Table 3) and survival (Fig. 3 and Supplementary Table S1) patterns, while explicitly stating that these analyses are meant to be hypothesis generating and cannot deliver definitive results due to the size and nature of the trial. Among pretreatment disease characteristics, we found that high-risk FISH cytogenetics (as defined by the presence of del17p and del11q) affected PFS (median PFS 46.4 months (95% CI 36.2–56.6) vs 70.5 months (95% CI incalculable) *p* = 0.037; HR 2.33 (95% CI 1.03–5.26) (Fig. 3c)). In addition, immunoglobulin variable region heavy chain (IgVH) mutation state affected PFS highly significantly (median PFS not reached vs 43.8 months (95% CI 35.3–52.3) *p* = 0·005; HR 3.9 (95% CI 1.41–10.80) (Fig. 3d)) and exerting a borderline effect on OS (median OS not reached *p* = 0.084; HR 5.27 (95% CI 0.64–43.13)). Interestingly, patients with mutated IgVH do particularly well in the long term, consistent with observations made in the context of FCR [28]. We found no other risk factors with significant negative prognostic impact on the level of PFS in our exploratory analyses. However, we observed a borderline significant effect of more complex genetic alterations as shown by a surrogate of two or more mutations found in our eight-gene panel (median PFS 39.1 months (95% CI 26.0–52.2) vs 70.5 (95% CI 53.2–87.3) *p* = 0·051; HR 3.35 (95% CI 1.00–0.07) Fig. 2e). Notably, we found fewer than expected progressions in patients with *SF3B1* mutations, although this observation was not statistically significant. On the level of OS, we found an expected strong effect of *TP53* mutation and effects of *NOTCH1* mutation and the presence of more than one mutation per patient from our gene set (Supplementary Table S1).

Central MRD assessment after the end of induction from peripheral blood was available in 39 of 40 patients evaluable for response. MRD negativity (defined as less than 10^−3^) [15] after induction treatment was achieved in 56.4% (22/39) of evaluable patients and was associated with a better median PFS (76.1 months (95% CI incalculable) vs 46.4 months (95% CI 33.5–59.3) *p* = 0·007)) (Fig. 3f).

### Analysis of potential predictive markers for aggregated lenalidomide toxicity

Our analyses of toxicities and individual MTDs suggested an individual component of tolerability for lenalidomide in combination with FR. About half of the dose-limiting events happened early and at very low doses of lenalidomide, suggesting a non-dose-dependent component to this group of events (Supplementary Fig. S2). In this group of patients, myelotoxicity was not a major contributor to dose limitation. While myelotoxicity was a seemingly dose-dependent contributor to the dose limit at higher doses of lenalidomide, at lower doses, we observed mainly skin toxicities; thrombosis (2 in the first 10 patients, none after amendment of aspirin prophylaxis) and patient’s wish to discontinue lenalidomide. The latter appeared to be a surrogate for more general symptoms the patients felt to be burdensome, but that could not be described by CTC criteria.

**Fig. 4:**
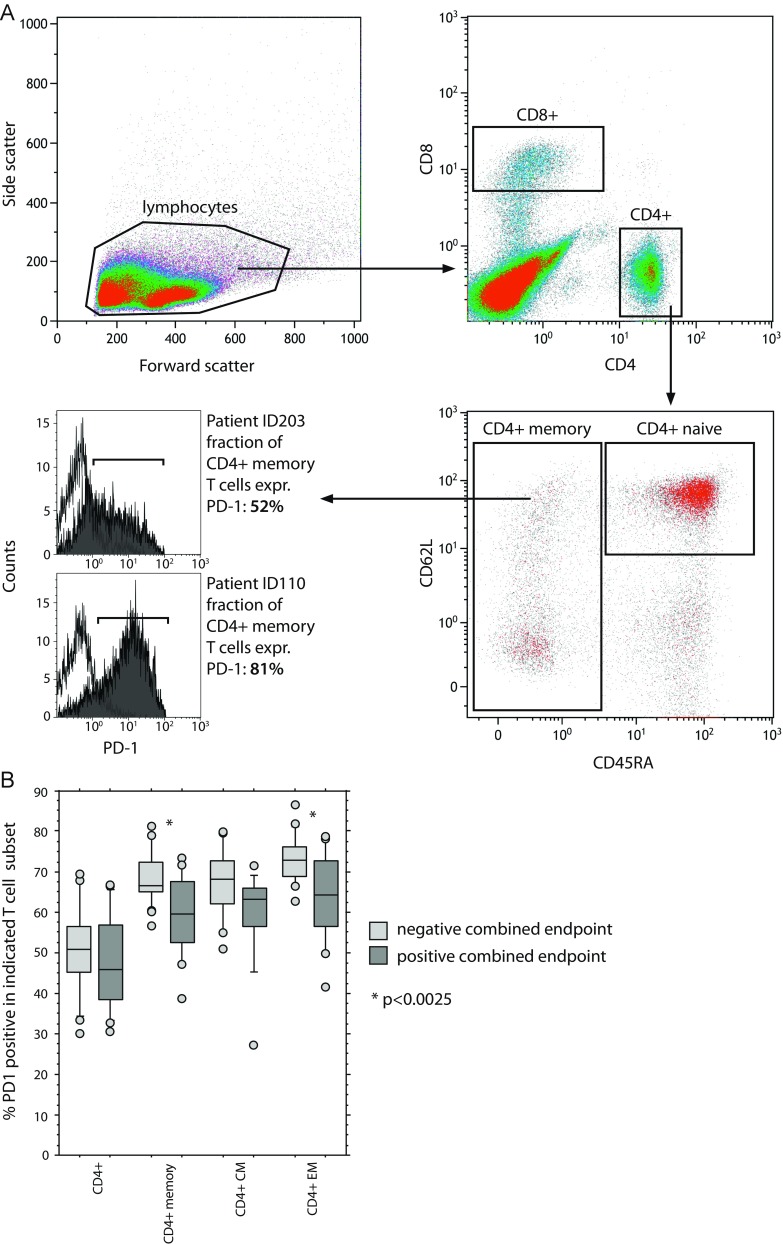
T cell subsets predict intolerance for higher lenalidomide doses. **a** Gating strategy to identify naïve and memory subsets and to measure PD-1 expression; **b** percentage of PD1-positive cells in CD4+ cells, CD 4 memory T cells and central and effector memory subgroups from pretreatment samples from 41 patients treated in the trial. Patients were categorised into two groups depending on whether they reached a composite endpoint of observed non-haematologic dose-limiting toxicities or inability to escalate dose beyond 5 mg lenalidomide

We had previously described that fludarabine treatment induces marked changes in the composition of the T cell compartment [29], leading to a more active T cell phenotype. Based on the pattern of side effects and the mechanism of action of lenalidomide in CLL (reviewed in Kater et al. [6]), we hypothesised that a T cell effect may determine these early dose-limiting events. We analysed T cell subsets and their state of exhaustion (Fig. 4a) in pretreatment samples. We defined a composite clinical endpoint, identifying patients that had either achieved a maximum tolerated dose of lenalidomide of 5 mg (or less) and/or had suffered any non-haematologic dose-limiting event. Using this binary marker, we were able to associate this group with T cell subsets and their exhaustion state. When applying a multiple testing correction, a significant association was found when analysing the exhaustion marker PD-1 in the CD4 memory T cell compartment (Fig. 4b), while total PD-1 positivity in the CD4 population was not different between groups. No similar association was observed for any of the CD8 T cell populations. In our analysis, a lower percentage of exhausted CD4 memory cells, specifically CD4 central memory cells, before treatment was highly associated with our composite endpoint surrogate for problems with lenalidomide dose escalation in induction treatment. By contrast, PD-1 positive memory CD4 cells were not associated with either response or PFS.

## Discussion

Combination chemoimmunotherapy remains a standard of care in first-line treatment of CLL [30, 31] and a randomised comparison between FCR and bendamustin/rituximab (BR) suggests that FCR is still the benchmark for the treatment of fit, untreated CLL patients [32]. However, recent years have seen attempts at integrating novel targeted drugs into such chemoimmunotherapy regimens. Most recently, combinations of bendamustine and rituximab with the kinase inhibitors ibrutinib and idelalisib were presented [33, 34]. In the case of idelalisib, the added toxicity presented a relevant problem for the patients and their management and in respect to both trials, there is an ongoing discussion about what the added value of the chemotherapy on top of the kinase inhibitor may be. The integration of novel drugs into combination regimens thus remains a major challenge. We aimed to define the feasibility of integrating the immunomodulatory drug lenalidomide into a chemoimmunotherapy regimen.

We found that in combination, chemoimmunotherapy and lenalidomide can be safely dose escalated to 10 mg or above in 65% of the patients. The combination of FR with lenalidomide was effective in terms of inducing responses and the overall strategy yielded a long PFS. In fact, the presented median PFS of 60 months compares very well with the 57 months reported for the FCR arm in the long-term observation of the German CLL8 trial [35], despite a somewhat more comorbid population. Thus, the combination of chemoimmunotherapy replacing cyclophosphamide by lenalidomide yielded an encouraging disease control. We also demonstrate an effective control of expected lenalidomide-associated problems with tumour lysis and flare phenomena in the cohort. In our experience, the maintenance phase with the rituximab/lenalidomide combination led to a limited improvement in responses (five patients (12% of the maintenance population) improved their clinical response during maintenance (from PR to CR)), but this showed significant myelotoxicity. We conclude that the maintenance regimen, based on the established dose from the dose escalation part of the trial, was too ambitious, and suggest that maintenance may be more efficient at lower, more tolerable doses, which is in line with the observed doses achievable in two randomised lenalidomide maintenance trials recently presented [36, 37].

In exploratory analyses, we found that achieving an MRD-negative state after induction was predictive of longer PFS, as has been reported for a number of other treatment modalities [38–40]. Similar to most reported chemoimmunotherapy experiences, we detected a significant difference in outcome by IgVH mutation state. IgVH-mutated cases had a particularly long PFS with a majority of patients remaining in remission past the 5-year mark. In addition, high-risk cytogenetics remained a negative prognostic factor, although the impression is that the negative effect may be smaller than in previously reported chemoimmunotherapy trials with FCR or BR [2, 41]. Despite the limitations inherent to a phase II trial, we aimed to analyse outcomes by mutations in a distinct set of eight genes that had been reported to affect outcomes in large sequencing studies [23–26] to present hypothesis generating findings. *TP53* and *NOTCH1* were associated with the strongest risk for reduced overall survival, and the combined presence of more than one of these eight mutations also proved as negative factor in this respect (Supplementary Table S1).

In our mind, the most surprising findings concern the pattern of toxicities, which we observed in our cohort. While in the majority of patients the major dose-limiting effect was a seemingly dose-dependent myelotoxicity, a subgroup of 35% did not tolerate doses above 5 mg lenalidomide throughout induction. In the respective patients, this became apparent very early on and at the lowest doses. Compared with 33% of patients that tolerated 25 mg lenalidomide, a dose up to tenfold of these patients, this phenomenon suggested an individual predisposition for a non-dose-dependent pattern of toxicities (Supplementary Fig. S2). Hereby, the most prominent toxicities were skin toxicities, also contributing the largest cause of dose limitation or study termination by the patients.

Our results may contain some more general lessons for the development of combination approaches with novel substances. Some potential lessons stem from the comparison of our trial with a trial performed in parallel at the Dana Farber Cancer Institute. Brown et al. [42] reported a trial with a highly similar treatment design but two potentially crucial differences: first lenalidomide was started at day 1 of the first cycle (together with the chemoimmunotherapy) and second, the trial was performed in a 3 + 3 design. The trial had to be stopped after nine patients at dose level − 1 (i.e. 2.5 mg every other day) for toxicity and futility reasons. How can we explain the fact that two thirds of our patients could tolerate more than five times the dose determined as a MTD in the trial by Brown et al.? We would like to propose two explanations: an argument of scheduling and one of trial design (individual dose escalation vs 3 + 3 design). A similar rate of early skin toxicities has not been reported in FR-treated or in lenalidomide monotherapy patients, suggesting a specific interaction of lenalidomide with the FR backbone in this respect. Since our previous work suggested significant changes in T cell reactivity early after fludarabine treatment, we speculate that a concomitant start of lenalidomide and fludarabine may lead to T cell-mediated toxic effects. Such effects may be mitigated by a delay in the start of the lenalidomide treatment, as was hypothesised in our trial design, since the parallel T cell depletion by fludarabine may limit such effects. Interestingly, a similar trial by Flinn et al., presented as meeting abstracts [43, 44], also showed overall tolerability of a combination of lenalidomide with FR and also suggested using a 1-week delay before adding lenalidomide. Confirming our experience, the authors also found rashes to be a main characteristic side effect of the combination. Another small trial, introducing lenalidomide in addition to a dose-reduced FCR regimen, also opted to start lenalidomide treatment at day 8 of the first cycle and reported on 20 evaluable patients [45]. Close to 5% of the patients had a grade 3/4 skin reaction and the authors described the overall toxicity profile acceptable. Comparing these results to those from Brown et al., we thus believe that the sequencing of the components of novel drug combinations may propose significant challenges for the development.

A second aspect relates to the trial design. Our experience suggested that a subpopulation of patients may have a predilection to suffer early non-dose-dependent toxicities leading to dose limitation and this group was significant in size. It is easy to see how such behaviour of a population could lead to a dismal outcome in a 3 + 3 design, since a relatively likely chance-recruitment of two patients from such a population may stop the dose escalation early and lead to a frustrating trial experience after a short interval. By comparison, our design of individual dose escalation in the first 10 patients allowed us to recognise the pattern, amend the trial accordingly and finish the trial with the presented results. We present these speculative interpretations as a note of caution regarding trial designs for substances that may have very complex mechanisms of action like lenalidomide and a potential to have significant toxicities in relevant subgroups. This may be especially important for combination approaches.

Finally, we asked ourselves whether we could identify such a subpopulation with increased toxicity upfront. We speculated that the T cell compartment of patients’ pretreatment may contain relevant information to this end. This was based on our earlier observations of T cell changes after fludarabine treatment [29], the fact that the mode of action of lenalidomide is thought to include relevant modulation of T cell function [6, 29, 46, 47] and the observation of excess skin toxicity, a phenomenon often associated with T cell activities [48]. Indeed, studies on the original Imid, thalidomide, had shown that it changed the recruitment of T cells to skin lesions in the treatment of cutaneous sarcoidosis and leprosy [49, 50]. Regarding a potential mechanism of T cell activation, lenalidomide has been shown to modulate exhaustion phenotypes of T cells in CLL [46, 47, 51] and we hypothesised that a reversal of T cell exhaustion may be associated with the clinical side effects profile observed in the combination. In order to analyse such a phenomenon, we devised a panel of immunostains to define T cell subpopulations and their state of exhaustion via PD-1 staining and compared the individual patient data with a combined clinical endpoint, devised as a surrogate for intolerability. When analysing the dataset using a multiple testing correction, it became apparent that the PD-1 expression levels on the memory T cell fraction of the CD4 positive T cells contained predictive information. Indeed, while overall PD-1 expression on T cells was similar between patients with or without significant trouble with lenalidomide dose escalation, the best correlation was found with the fraction of PD-1 positive CD4 memory cells, a T cell subpopulation we had previously found to be important in CLL progression [52]. In our current trial, this T cell subset was, however, not associated with either response to treatment or PFS. Overall, we propose that, while the mechanism of the interaction currently remains unclear, such a marker population may be useful to select patients for lenalidomide combination regimens in the future. This may be of special interest since recently, a number of trials containing both lenalidomide and agents targeting PD-1 were put on hold due to observed toxicities. Given our observation from this trial, it might be useful to include similar T cell analyses in the workup of trials investigating lenalidomide combination treatments.

In summary, the addition of lenalidomide to an FR chemoimmunotherapy backbone seemed feasible and effective, given a relevant attention to individual tolerability profiles of patients. However, based on the recent advances in the field, including novel combinations with high efficacy, but still short follow-up, it is unlikely that lenalidomide chemoimmunotherapy combinations will have a significant role in the treatment paradigm of previously untreated CLL.

## Electronic supplementary material

ESM 1 (PDF 247 kb)
