# Supplementary material for: Fludarabine and rituximab with escalating doses of lenalidomide followed by lenalidomide/rituximab maintenance in previously untreated chronic lymphocytic leukaemia (CLL): the REVLIRIT CLL-5 AGMT phase I/II study
Source: Ann Hematol. 2018 Jun 4;97(10):1825–39. doi: 10.1007/s00277-018-3380-z (PMC6097797; doi:10.1007/s00277-018-3380-z)
Supplement: Supplementary file 1 — (PDF 247 kb) [file 277_2018_3380_MOESM1_ESM.pdf]

## Supporting information

The file contains a table with statistical analyses showing effects of analysed risk factors on survival endpoints, it also contains a schematic depiction of the trial treatment for better illustration and a graph showing the timing of occurrence of dose limiting toxicities, showing the early dominance of non-hematologic toxicities.

*Supplementary table*

Table S1.

| | PFS | | | OS | | |
|---|---|---|---|---|---|---|
| | Exp(B) | 95% CI | p value* | Exp(B) | 95% CI | p value* |
| Gene mutations | | | | | | |
| *ATM* (yes vs no) | 1·86 | 0·80-4·34 | 0·14 | 0·80 | 0·21-2·99 | 0·74 |
| *BIRC3* (yes vs no) | 2·43 | 0·56-10·54 | 0·22 | 2·52 | 0·31-20·42 | 0·37 |
| *NFKBIE* (yes vs no) | 1·31 | 0·39-4·43 | 0·66 | 2·84 | 0·71-11·35 | 0·12 |
| *NOTCH1* (yes vs no) | 2·03 | 0·88-4·72 | 0·092 | 3·75 | 1·00-14·02 | 0·035 |
| *SF3B1* (yes vs no) | 0·41 | 0·06-3·05 | 0·37 | 0·97 | 0·12-7·80 | 0·98 |
| *TP53* (yes vs no) | 2·39 | 0·55-10·32 | 0·23 | 13·55 | 2·40-76·63 | <0·0001 |
| *ZNF292* (yes vs no) | 1·10 | 0·33-3·74 | 0·87 | 1·91 | 0·39-9·29 | 0·42 |
| FISH cytogenetics (high risk vs low risk)** | 2·33 | 1·03-5·26 | 0·037 | 1·94 | 0·25-7·30 | 0·32 |
| IgVH (unmutated vs mutated) | 3·90 | 1·41-10·80 | 0·005 | 5·27 | 0·64-43·13 | 0·084 |
| ≥2 mutations (yes vs no) | 3·35 | 1·00-0·07 | 0·051 | 4·06 | 1·01-16·26 | 0·032 |
| MRD neg after induction (yes vs no) | 0·33 | 0·14-0·77 | 0·007 | 0·53 | 0·12-2·39 | 0·40 |

*(Log Rank (Mantel-Cox))
** high risk defined by presence of del 17p and/or del11q, low risk defined as non-high risk

*Supplementary figures*

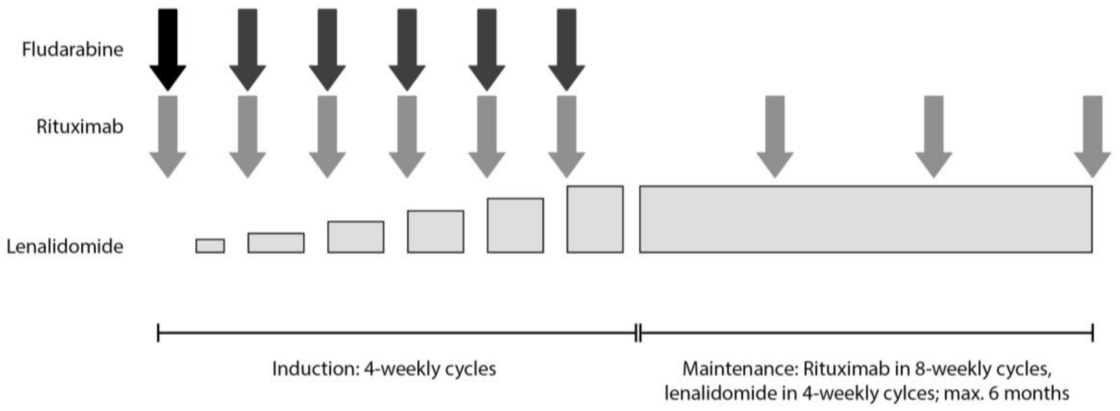

**Figure S1.** Trial design.

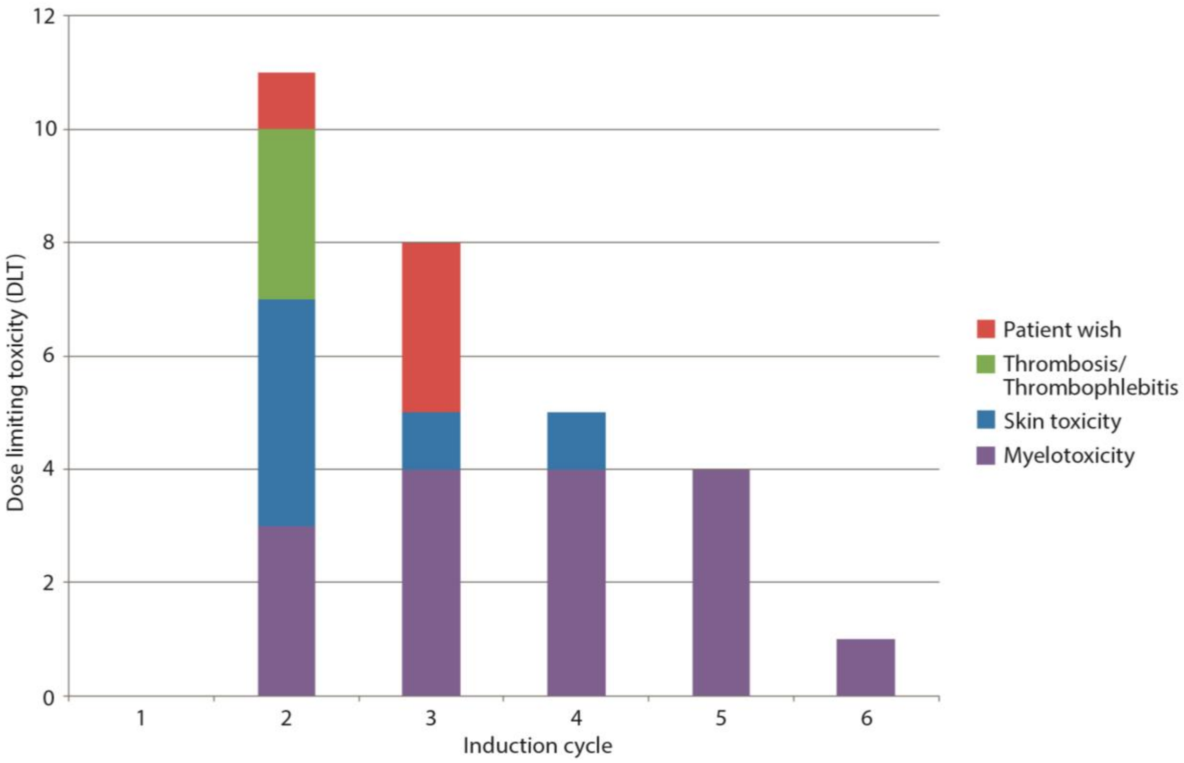


**Figure S2.** Occurrence of first dose limiting toxicity (DLT) per patient, as assessed at the end of treatment cycle (induction).
